# Spatial Profiling of Gingerol and Shogaol Analogues in Intact *Zingiber officinale* Rhizomes Using MALDI Mass Spectrometry Imaging

**DOI:** 10.3390/molecules31040618

**Published:** 2026-02-10

**Authors:** Josie C. Torrecampo, Neaven Bon Joy M. Marcial, Chuckcris P. Tenebro, Janine J. Salcepuedes, Paul Felipe S. Cruz, Phil Aidan C. Cruz, Jonel P. Saludes, Doralyn S. Dalisay

**Affiliations:** 1 Center for Chemical Biology and Biotechnology, University of San Agustin, Iloilo City 5000, Philippines; jtorrecampo@usa.edu.ph (J.C.T.); nbjmarcial@usa.edu.ph (N.B.J.M.M.); ctenebro@usa.edu.ph (C.P.T.); jsalcepuedes@usa.edu.ph (J.J.S.); 2 Herbanext Laboratories, Inc., Negros Occidental, Bago City 6101, Philippines; philipcruz.herbanext@gmail.com (P.F.S.C.); aidancruz.herbanext@gmail.com (P.A.C.C.); 3 Center for Natural Drug Discovery and Development, University of San Agustin, Iloilo City 5000, Philippines; jsaludes@usa.edu.ph; 4 Department of Chemistry, University of San Agustin, Iloilo City 5000, Philippines; 5 Department of Biology, University of San Agustin, Iloilo City 5000, Philippines

**Keywords:** MALDI mass spectrometry imaging, UPLC-ESI-QTOF-MS, ion mobility spectrometry, gingerol, shogaol, *Zingiber officinale*

## Abstract

Ginger (*Zingiber officinale*) is a widely recognized functional food, known for its anti-inflammatory, antioxidant, and digestive health benefits largely attributed to gingerol-related compounds. While traditional extraction-based methods have been used to characterize these metabolites, they often compromise the spatial context within tissues. This study represents the first application of matrix-assisted laser desorption/ionization mass spectrometry imaging (MALDI MSI) with ion mobility spectrometry (IMS) to map the detailed spatial distribution of key ginger metabolites (6-, 8-, and 10-gingerols and shogaols) in a complex matrix of an intact rhizome tissue. Rhizomes from five ginger accessions collected in Negros Occidental, Philippines, were cryosectioned at 20 μm, coated with 2,5-dihydroxybenzoic acid (DHB) matrix, and analyzed using MALDI MSI at 100 µm spatial resolution across an *m*/*z* range of 50–1200. The MALDI MSI revealed that 6-, 8-, and 10-gingerols were predominantly localized in the stele and cortex regions, while shogaols exhibited broader distribution, including the epidermis. Principal component analysis (PCA) on UPLC-ESI-QTOF-MS data of methanolic rhizome extracts revealed clustering patterns among the five ginger accessions. These findings provide a spatially resolved metabolomic profile of gingerols and shogaols, offering novel insights into the anatomical localization of bioactive compounds. This integrative approach establishes a foundation for future studies on ginger physiology, breeding, and quality control of ginger-derived natural products.

## 1. Introduction

Ginger (*Zingiber officinale*) is widely recognized not only as a culinary spice but also as a medicinal plant with applications in both traditional and modern medicine. This broad recognition has prompted scientific interest in exploring the phytochemical complexity of ginger and related species within the *Zingiberaceae* family, particularly through advanced analytical techniques. Comparative MS-based metabolomics studies of *Zingiberaceae* rhizomes, including ginger, galangal, and turmeric, using untargeted LC-MS combined with molecular networking and chemometrics, have shown that ginger possesses a distinctive chemical profile. In this analysis, specific metabolites including gingerol and shogaol analogues were highlighted as potential contributors to in vitro antidiabetic activity [1]. Its therapeutic potential is largely attributed to its phenolic constituents, particularly gingerols and their thermally derived counterparts, shogaols [2]. These metabolites have been extensively documented for their diverse biological properties, including anti-inflammatory, antioxidant, anticancer, neuroprotective, and gastrointestinal effects.

Extending from this molecular and biological evidence, clinical and preclinical investigations have substantiated the therapeutic potential of ginger supplementation, particularly in modulating inflammatory pathways and gastrointestinal function. In patients with active rheumatoid arthritis, daily administration of 1.5 g ginger over 12 weeks significantly reduced systemic inflammatory biomarkers, including interleukin-1β (IL-1β) and high-sensitivity C-reactive protein (hs-CRP) [3]. In oncology settings, ginger intake improved gastric myoelectrical activity, with participants reporting relief from symptoms such as nausea, dysmotility, and reflux [4]. Moreover, ginger-derived compounds exhibit neuroprotective activity relevant to neurodegenerative disorders, including Alzheimer’s and Parkinson’s diseases, by enhancing cognitive performance, promoting synaptogenesis, and safeguarding neuronal integrity. These effects are mediated through activation of signaling cascades such as ERK/CREB and Nrf2, and suppression of NF-κB, proinflammatory cytokines, and reactive oxygen/nitrogen species [5].

To better understand the mechanistic basis of these therapeutic effects, pharmacokinetic studies have explored the absorption and distribution of key bioactive compounds, particularly shogaols and gingerols. In rodent models, shogaols demonstrated faster absorption and longer half-lives compared to their corresponding gingerols, indicating a potentially greater contribution to in vivo bioactivity despite their lower concentrations in raw ginger. Furthermore, metabolites such as 6-shogaol and zingerone were detected in multiple tissues, including the brain, underscoring their capacity for systemic distribution and supporting the relevance of observed metabolite localization patterns within the rhizome [6].

Insight into the evolutionary context of gingerol and shogaol may provide a deeper understanding of the observed histological distribution of these compounds in planta. Gingerols and shogaols are classified as phenolic compounds structurally related to phenylpropanoids, which are believed to originate from amino acids produced via the shikimate pathway [7]. This process is well conserved across plant taxa, and is known to function primarily in the development of plant resistance to biotic and abiotic stress [7]. In the case of *Z. officinale*, these phenylpropanoids are largely modified by type III polyketide synthases (PKSs) coupled with NADPH-dependent alkanal/one oxidoreductases (AORs) to form highly lipophilic compounds that occur in the plant as an oleoresin [8,9].

Ginger oleoresin and gingerol have demonstrated antifungal, antimicrobial, and fungal-amylase inhibitory activities [10,11,12], in addition to antifeedant and insect growth-regulatory properties [13], supporting their functional role in plant defense and protection against herbivory. The widespread accumulation of gingerol in rhizomatic tissues, which serve as primary sites of starch storage, suggests an adaptive strategy for localized chemical defense [14]. To investigate the regulatory mechanisms underlying gingerol biosynthesis, transcriptomic and metabolomic analyses have been conducted. The reference ginger genome (accession PRJNA477462) revealed differential expression of genes associated with gingerol and diarylheptanoid biosynthesis, predominantly in the rhizome [9,15]. Sequencing studies identified gene families and transcription factors implicated in this pathway. Biosynthesis initiates with the conversion of L-phenylalanine to feruloyl-CoA, followed by downstream synthesis of gingerols [16]. Key differentially expressed genes (DEGs), including caffeoyl-CoA O-methyltransferase (CCoAOMT) and hydroxycinnamoyl-CoA shikimate/quinate hydroxycinnamoyl transferase (HCT), have been proposed as regulatory nodes in the production of gingerol metabolites [9].

Advances in analytical technologies have further enabled the detailed characterization of plant metabolites, including gingerol, by improving spatial resolution and structural identification in complex biological tissues. The integration of ion mobility spectrometry (IMS) into MALDI workflows has enhanced molecular imaging by adding an orthogonal gas-phase separation step. This additional dimension helps distinguish isobaric and isomeric metabolites, thereby improving annotation confidence. An application of this approach demonstrated that coupling IMS with MALDI MSI enabled the spatial separation of disaccharide isomers (trehalose and sucrose) within plant root tissues, highlighting its effectiveness for resolving structurally similar metabolites in situ [17]. Aside from imaging workflows, ion mobility spectrometry also provided an extra dimension of separation in other platforms. In 2D-LC/IM-QTOF-MS of ginseng, it enabled differentiation of over 300 ginsenosides and increased identification confidence [18], while in HS-GC-IMS of jujube, it allowed the detection of 47 volatile compounds including low-abundance and previously unreported sulfur-containing volatiles demonstrating its broad applicability and high resolving power for both non-volatile and volatile plant metabolites [19].

Despite extensive phytochemical research on *Z. officinale*, most studies focus on solvent-extracted metabolite profiles, often overlooking the spatial organization of bioactive compounds within the rhizome tissue. This limitation restricts our understanding of how specialized metabolites like gingerols and shogaols are distributed in relation to the rhizome’s anatomical features, which could have implications for biosynthesis, storage, and quality assessment. Histological and biological studies have suggested that oil cells and parenchymal tissues in the cortex and stele are the major sites of accumulation for lipophilic phenolic compounds [20,21,22,23], such as gingerols and shogaols. However, these findings are largely based on indirect methods, such as histochemical staining and tissue-specific assays, which do not provide definitive spatial resolution at the molecular level [24]. An emerging solution to this limitation is matrix-assisted laser desorption/ionization mass spectrometry imaging (MALDI MSI). This established analytical technique is used for visualizing plant metabolites in situ. For example, MALDI MSI revealed the spatiotemporal distribution of a natural HMG-CoA reductase (HMGCR) inhibitor in pineapple fruit tissue [25]. The technique was also able to map the lignans and cyanogenic glucosides across flax seed development [26].

To address the gap in tissue-level metabolite organization, we mapped metabolites in fresh, intact ginger rhizomes from five Visayan accessions (Philippines). This study aims to (i) visualize the spatial distributions of 6-, 8-, and 10-gingerols and their shogaols, (ii) correlate these localization patterns with rhizome microanatomy and inter-accession chemical diversity, and (iii) perform untargeted UPLC-QTOF-MS metabolomics with principal component analysis (PCA) to capture global variance across accessions and reveal natural sample groupings. We employed high-resolution MALDI MSI with ion mobility spectrometry and orthogonal UPLC-QTOF-MS and MS/MS validation. Collectively, this integrative spatial-untargeted metabolomics workflow generates a tissue-resolved atlas of gingerols and shogaols in Visayan ginger, links metabolite localization to rhizome microanatomy, and defines chemotypic signatures across accessions.

## 2. Results

### 2.1. Spatial Distribution of Gingerols and Shogaols in Ginger Rhizome

**Figure 1:**
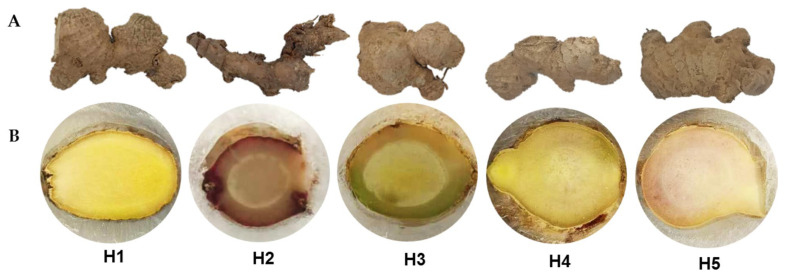
Five *Z. officinale* accessions collected from Negros Occidental, Philippines coded as H1, H2, H3, H4, and H5 highlighting both their (**A**) external rhizome morphology and the (**B**) internal cross-sections following tissue embedding.

MALDI MSI enabled the direct visualization of gingerols and shogaols within a complex matrix of intact tissue sections of ginger rhizomes, preserving spatial information across histological regions. In this study, we present the first MALDI MSI analysis of cross-sections of ginger rhizomes from five ginger accessions cultivated in the Visayan region of the Philippines (Figure 1). Visual inspection of the five ginger accessions following embedding revealed variation among accessions in both internal coloration and anatomical regions. Ginger H1 exhibited a bright yellow interior with reduced contrast between the stele and cortex, resulting in a more homogeneous internal structure. Ginger H2 showed a reddish-purple pigmentation with a clearly defined central stele and surrounding cortex. Ginger H3 displayed a pale yellow-green interior with well-distinguished stele, cortex, and epidermal regions. In contrast, ginger H4 showed a light-yellow interior with less apparent internal boundaries. Ginger H5 exhibited a pinkish outer region where the epidermis and cortex were also less apparent.

**Figure 2:**
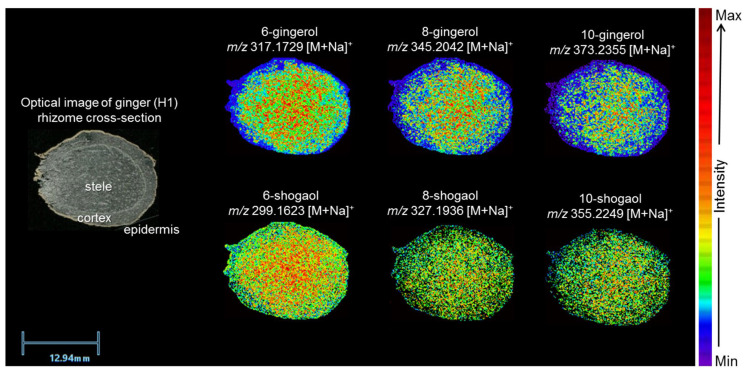
Optical image of a tissue section from *Z. officinale* H1 annotated with epidermis, cortex, and stele regions, alongside MALDI ion images showing the spatial distribution of sodiated adducts ([M+Na]^+^) of 6-, 8-, and 10-gingerols and shogaols. Ion images are displayed on a normalized heatmap scale (red = 100%, violet = 0%) with tissue thickness of 20 µm, spatial resolution of 100 µm, and a scalebar of 12.94 mm.

To investigate the spatial localization of major bioactive compounds in ginger, MALDI MSI was performed on cross-sections of ginger rhizomes. The optical image of a representative rhizome (H1) clearly delineates key anatomical regions, including the epidermis, cortex, and stele, providing a structural framework for interpreting metabolite distribution (Figure 2). The anatomical differences observed among the five ginger accessions are consistent with known natural variability in *Z. officinale*. Ginger varieties differ significantly in rhizome color, cortex-to-stele ratio, vascular arrangement, and parenchymal pigmentation due to genetic diversity, rhizome maturity, and environmental growing conditions. Genotype-dependent differences in storage tissue development, lignification, and phenolic accumulation have been documented across ginger cultivars. Environmental factors such as soil nutrients, moisture, and cultivation practices can further influence rhizome morphology and color, contributing to the variation seen among accessions. These combined genetic and environmental effects likely explain the distinct anatomical features observed in Figure 1.

Gingerols and shogaols predominantly appeared as sodiated ([M+Na]^+^) adducts in the MALDI MSI data, reflecting the intrinsic sodium present in plant tissues, which is naturally absorbed and distributed for osmotic regulation and ion homeostasis [27]. Small hydroxyl- or carbonyl-containing metabolites often form sodium or potassium adducts under positive ion MALDI conditions. MALDI MSI ion maps of sodium adducts ([M+Na]^+^) corresponding to 6-, 8-, and 10-gingerols and their respective shogaols (6-, 8-, and 10-shogaol) revealed widespread yet heterogeneous distributions throughout the rhizome tissue (Figure 2). Rather than being confined to distinct anatomical zones, the spatial patterns of these compounds were broadly overlapping, lacking sharp regional compartmentalization. Among the gingerols, 6-gingerol (*m*/*z* 317.1729) displayed a non-uniform and highly intense signal, with the highest accumulation observed in the inner cortex and stele. 8-gingerol (*m*/*z* 345.2042) exhibited a similar but slightly more localized distribution, particularly in the central stele. In contrast, 10-gingerol (*m*/*z* 373.2355) showed a diffuse and discontinuous signal, with generally lower abundance and no strong enrichment in any specific tissue region.

The shogaol analogs followed comparable yet slightly more uniform spatial trends. 6-shogaol (*m*/*z* 299.1623) was among the most intense signals observed, displaying widespread and more evenly dispersed signals across the tissue, including the epidermis. Meanwhile, 8-shogaol (*m*/*z* 327.1936) demonstrated a nearly uniform gradient from cortex to epidermis, suggesting systemic accumulation. 10-shogaol (*m*/*z* 355.2249) also followed a broadly distributed pattern, with slightly elevated signals in the central stele.

**Figure 3:**
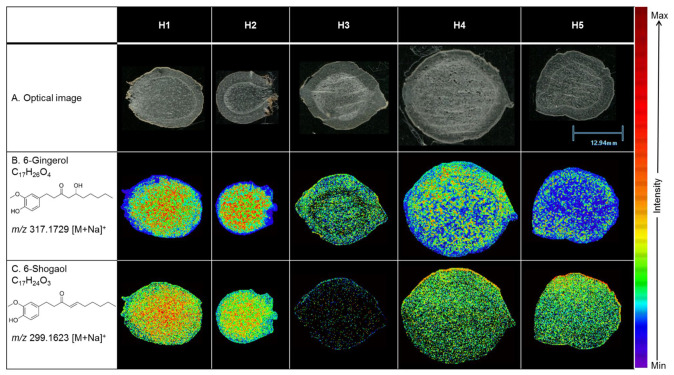
(**A**) Optical image of a tissue section from *Z. officinale* H1–H5. MALDI ion images comparing five *Z. officinale* accessions (H1–H5). (**B**) Distribution of 6-gingerol [M+Na]^+^. (**C**) Distribution of 6-shogaol [M+Na]^+^. Ion images are normalized to total ion count, displayed in heatmap scale (red = 100%, violet = 0%), with tissue thickness of 20 µm, spatial resolution of 100 µm, and a scalebar of 12.94 mm.

To assess whether the observed patterns in H1 are conserved across genetic backgrounds, the spatial distributions of 6-gingerol and 6-shogaol were analyzed in five ginger accessions (H1–H5) using the same MALDI MSI approach (Figure 3). In Figure 3B, 6-gingerol distribution varied considerably across accessions. H1 and H2 exhibited relatively high signal intensity and broad coverage, particularly within the cortex and stele. H3 showed low intensity with sparse, discontinuous patches, while H4 displayed a moderate but widespread signal. H5 presented the lowest overall abundance, limited to a few localized regions. Similar spatial patterns were observed for 8-gingerol, 10-gingerol, and their corresponding shogaols (Figure S1).

In contrast, Figure 3C shows the distribution of 6-shogaol, which remained consistently detectable and broadly dispersed across all five accessions. Even in accession like H3, where 6-gingerol levels were minimal, 6-shogaol exhibited a relatively uniform and spatially continuous signal. Although 6-shogaol was detected in H3 based on accurate mass and ion mobility drift time alignment with the reference standard (Table S1), the signal appears faint in the ion image due to its low ion abundance relative to the fixed heatmap scale. Thus, the apparent lack of a visible feature in the figure reflects very low intensity and not attributed to the absence of the metabolite. Similar spatial patterns were observed for 8-gingerol, 10-gingerol, and their corresponding shogaols (Figure S1). These inter-accession comparisons reinforce the trend observed in H1: gingerols exhibit greater spatial heterogeneity and variability in abundance, while shogaols are more uniformly distributed, both within and across accessions.

### 2.2. Histological Mapping of Oil Cells

**Figure 4:**
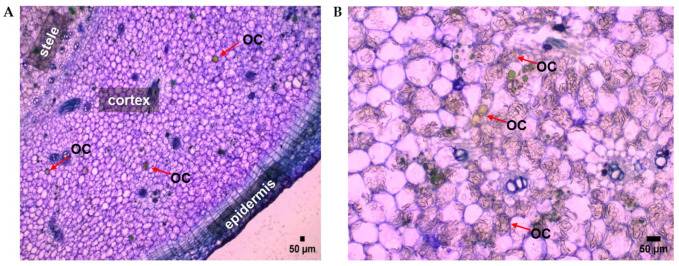
Histological architecture of TBO-stained ginger rhizome tissue and localization of oil cells (OC). (**A**) Lower magnification cross-section showing the epidermis, cortex, and stele. Oil cells are prominently distributed in the cortex and stele, with no visible presence in the epidermis. (**B**) High-magnification light microscopy image of the stele region showing large, spherical oil cells (indicated by red arrows). Scale bars: 50 µm.

To further understand the tissue-specific localization of gingerols and shogaols observed via MALDI MSI, histological analysis of ginger rhizome cross-sections was conducted using bright-field light microscopy. This approach aims to identify cellular structures potentially responsible for the biosynthesis, accumulation, or storage of secondary metabolites. The anatomical organization of the rhizome was clearly visualized, revealing three major tissue zones: epidermis, cortex, and stele (Figure 4).

The outermost epidermis appeared as a single protective cell layer, devoid of specialized secretory or storage structures. Beneath it, the cortex was composed of loosely packed parenchyma cells, interspersed with distinct spherical oil cells (OC). The stele, occupying the central region of the rhizome, was densely packed with parenchyma, rich in starch grains (SG), and contained numerous oil cells (Figure 4). The parenchyma cells exhibited high cytoplasmic density, consistent with their metabolic activity. In contrast, oil cells stood out due to their size and clear, vacuolated interior, typical of storage sites for lipophilic or phenolic compounds [22].

Oil cells were found to be abundant in the cortex and stele, but completely absent in the epidermis, a pattern consistently observed across multiple rhizome sections. These cells were morphologically distinct, featuring thick walls, large vacuoles, and clear intracellular space, indicative of storage compartments [22,28].

### 2.3. Orthogonal Validation of Gingerol and Shogaol Assignments by Ion Mobility and LC-MS Analysis

To ensure confident identification of gingerols and shogaols detected in rhizome cross-sections by MALDI MSI, orthogonal verification was carried out using MALDI-QTOF ion mobility spectrometry and UPLC-QTOF-MS analysis of authentic standards and methanolic ginger extracts. This multimodal approach was critical for resolving analytical challenges, particularly the *m/z* overlap between gingerols and their dehydrated shogaol counterparts, and for supporting accurate molecular assignments in complex tissue-derived spectra.

**Figure 5:**
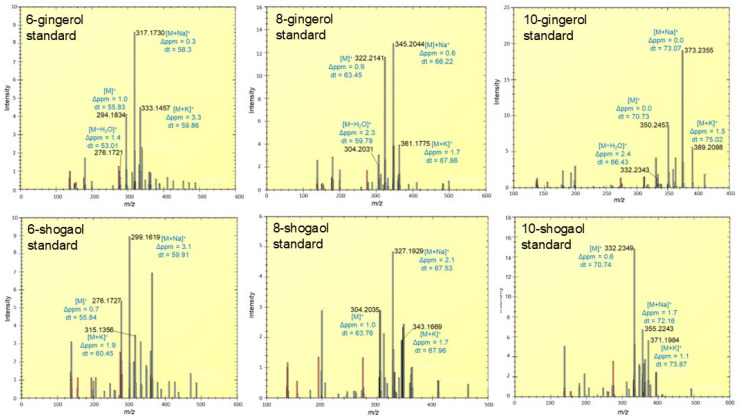
Representative MALDI-TOF mass spectra of pure gingerol and shogaol standards. The most prominent ion species of gingerols include [M]^+^, [M−H_2_O]^+^, [M+Na]^+^, and [M+K]^+^ adducts. In contrast, shogaols primarily yielded [M]^+^, [M+Na]^+^, and [M+K]^+^ ions. These spectral profiles serve as reference signatures for compound identification and spatial mapping in tissue-based MSI analyses. The peaks attributed to DHB are highlighted in red and correspond to the following matrix-related species: [DHB–H_2_O+H]^+^, [2DHB−2H_2_O+H]^+^, [DHB+K]^+^, and [DHB]^+^.

Ion mobility analysis of authentic standards revealed distinct ionization profiles for gingerols and shogaols under MALDI conditions. Gingerols predominantly produced [M]^+^, [M−H_2_O]^+^, and [M+Na]^+^ adduct ions, while shogaols generated [M]^+^ and [M+Na]^+^ species (Figure 5). In addition to the ions assigned to the target analytes, several auxiliary peaks were observed in the mass spectra. These signals are predominantly attributable to DHB matrix-related adducts, which are commonly formed during the MALDI ionization process [29]. A critical analytical complication arose due to the *m*/*z* equivalence between the [M−H_2_O]^+^ ion of gingerols and the [M]^+^ ion of the corresponding shogaols, which can lead to misidentification in conventional MS-based imaging. However, ion mobility spectrometry enabled baseline separation of these isobaric ions, as they exhibited distinct drift times, attributable to structural differences in molecular geometry and polarity. For example, drift time separation between 6-gingerol [M−H_2_O]^+^ and 6-shogaol [M]^+^ with drift time of 53.01 bin and 55.84 bin, respectively, confirmed that these ions, although identical in mass, are chemically distinct and independently detectable in MALDI MSI data. This confirmation ensures that MS imaging signals mapped to each compound accurately reflect the true chemical identity, rather than in-source artifacts or overlapping species. These results align with prior studies demonstrating the utility of ion mobility for resolving isobaric natural products in plant tissues. A notable example is the separation of the isobaric molecules ponasterone A and ecdysone, which share the same exact mass (*m*/*z* 487.3036 [M+Na]^+^) but exhibit distinct drift times allowing differentiation that would not be possible through mass analysis alone [30].

**Figure 6:**
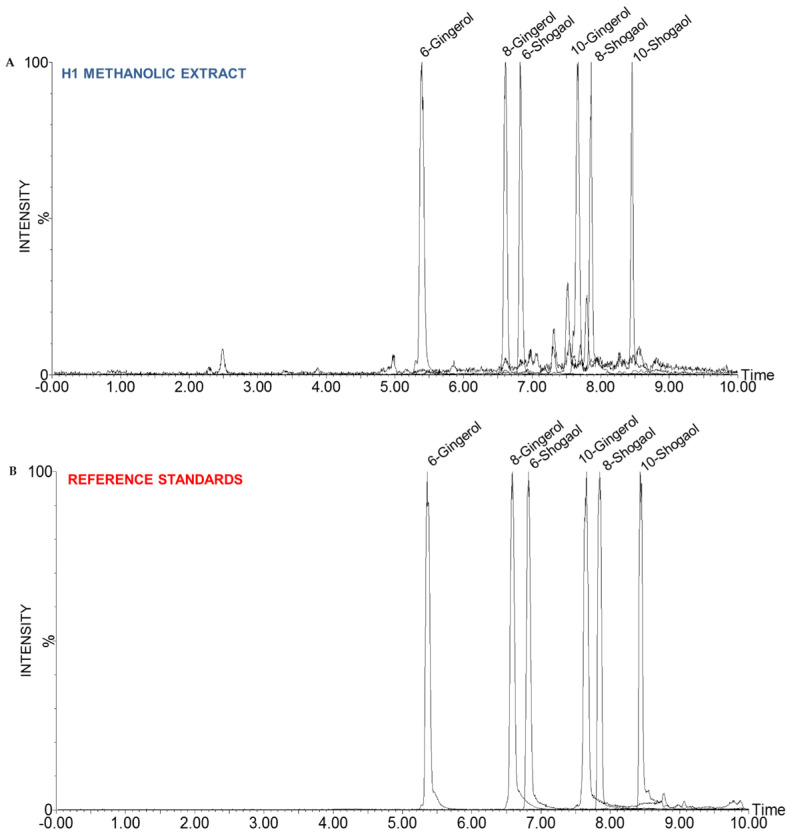
UPLC-ESI-QTOF-MS analysis of ginger methanolic extracts and reference standards in positive ion mode. (**A**) EICs of 6-gingerol, 8-gingerol, 10-gingerol, 6-shogaol, 8-shogaol, and 10-shogaol in methanolic extracts of ginger H1. (**B**) EICs of pure reference standards analyzed under identical conditions. Retention time alignment between extract and standards confirms compound identity and supports downstream MSI data interpretation.

To further confirm molecular identity and retention characteristics, UPLC-QTOF-MS was performed on both authentic standards and methanolic extracts of *Z. officinale* accession H1 (Figure 6). All six target compounds, 6-, 8-, and 10-gingerol, and 6-, 8-, and 10-shogaol, were successfully detected in the methanolic extracts, exhibiting elution patterns consistent with polarity, i.e., gingerols eluted earlier than their more hydrophobic shogaol analogues. Gingerol standards primarily produced [M−H_2_O+H]^+^, [M+Na]^+^, and [2M+Na]^+^ ions, while shogaols yielded [M+H]^+^, [M+Na]^+^, and [2M+Na]^+^ (Figure S2). These patterns serve as diagnostic ion signatures for compound tracking in both extract and tissue-based analyses. In the methanolic ginger extracts, extracted ion chromatograms (EICs) confirmed the presence of all six compounds, with retention times matching those of the standards. The adduct profiles were consistent with the standards for gingerols, while for shogaols, the [M+Na]^+^ ion was the most consistently observed (Table S2).

To verify structural identity, tandem MS experiments were performed on [M+Na]^+^ ions of the four most abundant compounds (6-, 8-, 10-gingerol and 6-shogaol). The MS/MS spectra of 6-gingerol ([M+Na]^+^ at *m*/*z* 317) exhibited fragment ions at *m*/*z* 179 and 137, consistent with cleavage of the β-hydroxy-containing side chain and formation of the vanillyl cation, respectively. The *m*/*z* 179 fragment retains part of the side chain and is characteristic of gingerols. In contrast, 6-shogaol ([M+Na]^+^ at *m*/*z* 299) showed a dominant fragment at *m*/*z* 137 only, reflecting direct cleavage to the aromatic vanillyl cation due to the presence of an α,β-unsaturated ketone and absence of the β-hydroxy group (Figure S3). Similar fragmentation patterns were observed for 8- and 10-gingerol (Figure S4). On the other hand, while 8- and 10-shogaol were detected in EICs and showed correct retention alignment, their signal intensities were insufficient for reliable MS/MS fragmentation, indicating lower abundance or reduced ionization efficiency under the conditions used. Nonetheless, their chromatographic behavior and ion signatures provided sufficient confidence for tentative identification.

### 2.4. Inter-Accession Metabolite Patterns (LC-MS-Based)

**Figure 7:**
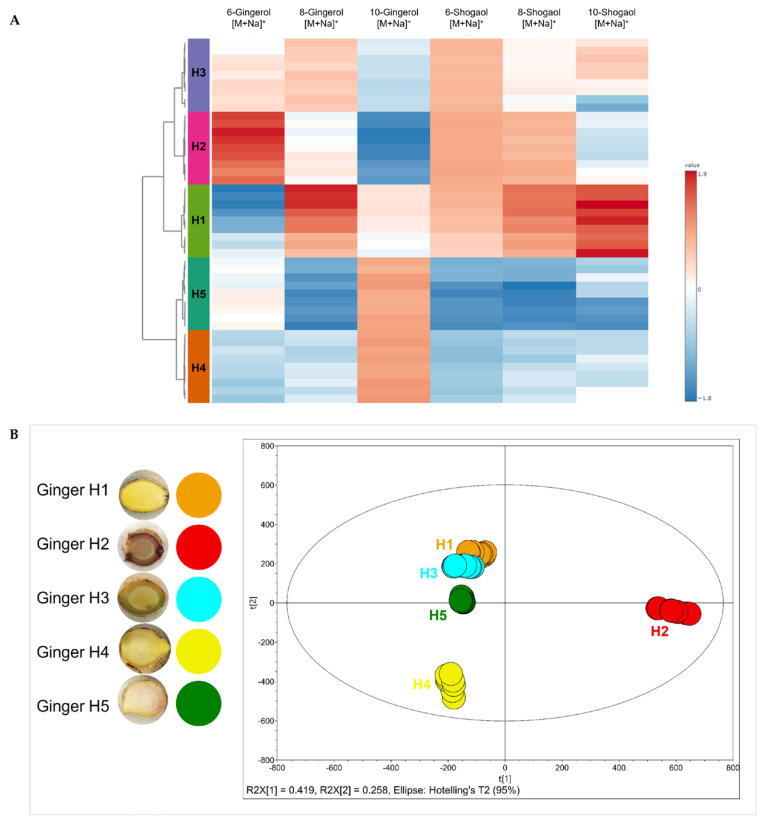
(**A**) Heatmap with hierarchical clustering analysis of 6-,8-, and 10-gingerols and shogaols detected as sodiated adducts [M+Na]^+^ in five ginger extracts. Data were normalized and processed using MetaboAnalyst, with color intensity representing relative abundance levels (red: higher, blue: lower). (**B**) PCA was based on UPLC-ESI-QTOF-MS-derived metabolite features (*n* = 9 per accession) filtered by ANOVA (*p* ≤ 0.05) and fold change ≥ 2, and Pareto scaled. The first two principal components (PC1 and PC2) explain 41.9% and 25.8% of the total variance, respectively.

To explore inter-accession differences in metabolite accumulation, methanolic extracts of rhizome tissue from five ginger accessions (H1–H5) were analyzed by UPLC-QTOF-MS. Signal intensities of [M+Na]^+^ adducts for six key metabolites, 6-, 8-, and 10-gingerol and 6-, 8-, and 10-shogaol, were normalized and visualized using hierarchical clustering and heatmap analysis (Figure 7A). Unlike tissue imaging, which resolves spatial localization, this LC-MS-based approach provides a global view of gingerol and shogaol abundance in each sample, integrating contributions from all tissue zones and cellular compartments. The heatmap revealed distinct chemotypic clusters. H1 in Cluster 1, a high-accumulating chemotype, was clearly distinguished from the other accessions, consistently exhibiting high levels of both gingerols and shogaols (Figure 7A). Notably, 6- 8- and 10-shogaol showed strong signals, along with elevated levels of 8-gingerol.

Accessions H2 and H3 formed Cluster II, an intermediate accumulating chemotype, marked by moderate levels of 6-gingerol and 6-shogaol, with reduced accumulation of longer-chain analogs like 10-gingerol and 10-shogaol (Figure 7A).

In contrast, accessions H4 and H5, which are low-accumulating chemotypes, were grouped into Cluster III, characterized by consistently low levels of all six metabolites, particularly the 6- and 8-carbon analogs (Figure 7A).

### 2.5. Principal Component Analysis

To evaluate broader, untargeted metabolic differences across ginger accessions, principal component analysis (PCA) was performed on UPLC-ESI-QTOF-MS data acquired from methanolic rhizome extracts of five *Z. officinale* accessions (H1–H5), each with nine biological replicates (*n* = 45 total). Unlike the targeted heatmap analysis in Figure 7A, this approach encompassed a comprehensive profile of detectable metabolite features, reflecting both known and unknown constituents. The resulting PCA model explained 67.7% of the total variance, with PC1 accounting for 41.9% and PC2 for 25.8% (Figure 7B).

The PCA score plot reveals a generally tight clustering of samples within each accession, indicating high biological consistency among replicates and reproducibility of extraction and analysis protocols. Despite this, notable separation between accessions is evident, indicating distinct global metabolic profiles. Accession H2 showed clear separation along PC1, indicating that it diverges the most in the dimension representing the largest variance (Figure 7B).

The PCA result aligns with heatmap data (Figure 7A), showing elevated levels of 6-gingerol and suggesting broader metabolic divergence beyond just targeted gingerol/shogaol compounds. The ginger H4, by contrast, is primarily separated along PC2, implying that it differs along a secondary axis of variation, possibly involving less abundant or structurally distinct metabolites (Figure 7B). This observation reinforces earlier findings of low gingerol/shogaol accumulation and suggests H4 may harbor a chemically distinct but low-abundance profile, potentially rich in unique minor metabolites not captured in targeted analyses. Accessions H1, H3, and H5 form an overlapping cluster near the center of the plot, indicating greater metabolic similarity (Figure 7B). This spatial proximity in the PCA model suggests shared biochemical traits or similar environmental or developmental influences. Interestingly, this contrasts with the heatmap analysis in which H1 was distinct due to high targeted metabolite content, highlighting how untargeted and targeted profiling provide complementary insights.

## 3. Discussion

The MALDI MSI revealed the spatial distribution of gingerols and shogaols in ginger rhizomes are heterogeneous, overlapping, and not anatomically distinct. The lack of strict tissue localization suggests that these metabolites may be synthesized across multiple cell types or transported between tissue zones.

The spatial disparity exhibited by gingerols and shogaols may be attributed, in part, to the localized expression of the KAT2 gene, a key enzyme in the peroxisomal β-oxidation pathway implicated in gingerol biosynthesis [31], resulting in tissue-specific accumulation patterns. Conversely, the broader distribution of shogaols may result from the non-enzymatic thermal conversion of gingerols, a process enhanced by environmental or post-harvest heat exposure [32], which could occur uniformly throughout the rhizome tissue, thus reducing spatial variability.

The spatial and accession-dependent distribution patterns of gingerols and shogaols, as revealed by MALDI MSI, underscore the non-compartmentalized, heterogeneous nature of gingerol and shogaol distribution in ginger rhizomes. The more variable and tissue-dependent distribution of 6-gingerol reflects its dynamic biosynthesis and susceptibility to environmental and processing influences, such as drying methods and polyphenol oxidase activity, which can modulate its accumulation and conversion [10]. In contrast, the more stable and uniform presence of 6-shogaol formed through enzymatic or thermal dehydration of gingerols, may also reflect developmental factors, as metabolite accumulation in *Zingiber* species has been shown to vary significantly across growth stages, with peak bioactive compound levels observed at maturity [33].

These findings provide new insights into the chemical architecture of ginger, supporting the hypothesis that gingerols and shogaols, although structurally related, may serve distinct physiological roles. From a practical standpoint, this spatial information is valuable for guiding optimized extraction strategies for pharmaceuticals or nutraceuticals and for identifying high-yield cultivars in breeding programs. Moreover, the application of MALDI MSI in this study highlights the power of spatial metabolomics to resolve fine-scale differences in compound localization that are not discernible through bulk analysis. A similar approach was used to identify the tissue-specific localization of asparaptine A in *Asparagus officinalis*, revealing spatial patterns that conventional methods could not resolve [34]. Such approaches enable a deeper understanding of tissue-specific metabolism, inter-accession chemical diversity, and the dynamic nature of specialized metabolite biosynthesis in medicinal plants.

The exclusive localization of oil cells within internal tissues aligns closely with the MALDI MSI results from Section 2.1 and Figure 2 and Figure 3, which showed that gingerols and shogaols accumulated preferentially in the cortex and stele, with minimal signal in the epidermis. This anatomical evidence suggests that oil cells function as primary storage sites for these bioactive metabolites. Given their structural characteristics and known role in essential oil accumulation in *Zingiberaceae* and other aromatic plants [20,21,22], these cells likely serve a dual role in both sequestration and protection of phenolic compounds such as gingerols and shogaols.

The observed tissue distribution of oil cells provides a compelling anatomical basis for the heterogeneous but cortex-stele-concentrated ion signals recorded by MALDI MSI. In particular, 6- and 8-gingerol, which showed enriched and patchy ion signals in the inner cortex and stele (Figure 2), correspond to the locations where oil cells were most dense. In addition, 6- and 8-shogaol, which had more evenly dispersed but still cortex-stele-biased distribution, align with both oil cells and metabolically active parenchyma capable of intercellular transport and storage. Further, minimal or absent signals for both compound classes in the epidermis correlate with the complete absence of oil cells in that tissue zone. These findings strongly support the hypothesis that the distribution of gingerols and shogaols is driven not only by biosynthesis but also by the presence of specialized storage structures, primarily oil cells. Previous work showed that signals for the pungent compounds like 6-gingerol were specifically traced to organelles containing yellow oil droplets, rather than being distributed throughout the tissue [35]. This supports our observations and suggests that gingerols preferentially accumulate in oil-rich compartments of the rhizome, underscoring the role of oil cells as key sites of metabolite localization.

While this study focused on a single accession (H1) for histological analysis, the conservation of cortex–stele metabolite accumulation across five ginger accessions (Section 2.1) suggests that oil cell localization may also be conserved and genetically regulated. This implies that differences in metabolite abundance across accessions (e.g., high 6-gingerol in H1 and H2 vs. low in H5) could reflect variation in oil cell density, metabolic capacity of adjacent parenchyma, or expression of key biosynthetic enzymes localized to specific cell types. In addition to serving as specialized storage compartments, oil cells in ginger rhizomes may interact with surrounding organelles such as the endoplasmic reticulum and mitochondria, potentially supporting localized metabolic processes involved in compound stabilization or secretion [22,36]. Their strategic location in the inner tissue zones may also help protect sensitive compounds from environmental degradation.

The integration of histological mapping with spatial metabolite imaging reveals a coherent picture of how the anatomy of ginger rhizome tissue supports and explains the observed chemical distributions. Oil cells and surrounding parenchyma are the primary anatomical correlates of gingerol and shogaol localization, confirming that spatial metabolic patterning is intimately linked to structural specialization in ginger. This dual-layer approach, combining chemical imaging using MALDI MSI and cellular histology, offers a robust framework for studying secondary metabolism in medicinal plants and highlights how specific cell types contribute to the compartmentalization and regulation of bioactive compound accumulation.

The orthogonal validation strategies provide robust confirmation of gingerol and shogaol assignments made via MALDI MSI, confirming that the spatial distribution patterns are not artifacts of ion overlap or fragmentation. The integration of ion mobility spectrometry, LC-MS, and MS/MS offers a validated workflow for metabolite localization studies in other medicinal plant species.

The heatmap analysis of the six key compounds showed that ginger H1 has the most abundant shogaol analogues. Shogaols, in particular, are associated with a range of bioactivities, including anti-inflammatory effects through HDAC inhibition and HSP70 induction [37], induction of autophagy in cancer cells via the AKT/mTOR signaling pathway [38], and suppression of cancer cell migration through inhibition of the IKKβ/NF-κB/Snail axis [39]. More recently, shogaol-enriched extracts have also demonstrated neuroprotective effects against oxidative stress-induced toxicity [40]. These findings further support the functional significance of shogaol accumulation and highlight the potential of H1 as a high-value cultivar for nutraceutical and therapeutic applications.

The high levels of 6-gingerol in H2, relative to its shogaol products, may reflect reduced thermal degradation or dehydration during post-harvest processing, consistent with previous findings that shogaol formation is primarily driven by heat-induced conversion of gingerols [10]. While not as rich in total content as H1, H2 and H3 may be favorable for selective extraction of shorter-chain gingerols, which have been reported to possess higher bioavailability compared to their longer-chain counterparts [6].

The low levels of gingerols and shogaols in H4 and H5 indicate a metabolically quiescent profile, potentially due to downregulation of key gingerol biosynthetic genes and lower accumulation of secondary metabolites in mature rhizome tissues [41].

These low-accumulating accessions may be of limited interest for pharmaceutical or high-value extract purposes but could serve as important controls or as a genetic basis for understanding pathway regulation. Their profiles also suggest susceptibility to post-harvest degradation, or differences in stress-induced metabolite synthesis, which is known to influence gingerol and shogaol levels.

This LC-MS-based chemotyping demonstrates that metabolite accumulation is not uniform across genotypes, even under standardized extraction and analysis conditions. Instead, accession differences reflect intrinsic genetic and metabolic factors, such as differential expression of biosynthetic enzymes such as cytochrome P450 oxidoreductases involved in secondary metabolism [42], variations in oil cell density or capacity [36], and differences in the post-synthetic stability of gingerols and shogaols under physicochemical conditions [43]. Importantly, the fact that shogaols often exceeded gingerols in abundance in H1 and H2 suggests that in planta conversion (via dehydration) may occur to varying degrees depending on genotype, maturity, or post-harvest handling. This supports the existing literature that emphasizes the dynamic relationship between gingerol content and its thermal or enzymatic conversion into shogaols during drying, aging, or metabolic stress [10,44,45].

The separation of H2 along the PC1 of the PCA score plot suggests a unique metabolomic signature, potentially driven by significant differences in geographic origin and environmental conditions such as altitude, soil properties, and nutrient availability [46,47]. The PCA results support the notion that ginger accessions differ not only in the absolute content of known bioactives but also in overall metabolome composition (Figure 7B), reflecting diverse genetic, biochemical, or ecological factors. The distinct positioning of H2 and H4 suggests these accessions may produce alternative classes of specialized metabolites, exhibit divergent expression of secondary metabolite pathways, or possess unique adaptive chemical traits under different growing or processing conditions. Such variation aligns with previous findings that metabolite profiles in *Z. officinale* are influenced by geographical origin and environmental conditions [46,48], as well as by underlying genetic and transcriptional differences, including regulation by biosynthetic genes [15] and long non-coding RNAs [48].

Moreover, the untargeted nature of this PCA analysis highlights the presence of potentially uncharacterized metabolic differences, which could be explored in future MS/MS-based metabolite identification workflows. Accessions like H2 and H4 may yield novel compounds of pharmacological interest, warranting deeper investigation through dereplication and structure elucidation techniques.

## 4. Materials and Methods

### 4.1. Plant Materials and Chemicals

Ginger rhizomes were supplied by Herbanext Laboratories Inc. (Bago City, Negros Occidental). Ginger H1 (CEBU2025101) and ginger H2 (CEBU2025102), sourced from Bago City. Ginger H3 was obtained from Don Salvador Benedicto (CEBU2025103), while ginger H4 (CEBU2025104) and ginger H5 (CEBU2025105) were collected from Ma-ao, Negros Occidental. All ginger samples were authenticated through expert identification by the resident botanist of the University of San Carlos and deposited in the university’s department herbarium. Reference standards, 6-, 8-, 10-gingerol, 6-, 8-, 10-shogaol were all purchased from ChemFaces (Wuhan, China). LC-MS grade methanol, acetonitrile, and formic acid were obtained from Thermo Fisher Scientific (Waltham, MA, USA).

### 4.2. MALDI-TOF MSI Sample Preparation

The freshly harvested ginger rhizomes were cut into sizes that could fit in a 6-well mold, embedded in a 3% agarose, and stored at −80 °C overnight [25,26,49,50]. Tissue embedding provides structural support and helps preserve tissue morphology. Samples were sectioned at 20 µm thickness using a LeicaCM 1950 cryostat (Leica Biosystems, Nussloch, Germany) with a maintained temperature of −14 °C. The ginger sections were thaw-mounted on clean MALDI plates and were dried in a desiccator cabinet for 30 mins prior to the matrix application. HTX M5 Sprayer (HTX Technologies, LLC, Carrboro, NC, USA) was used in matrix application. Ginger sections were coated with 2,5-dihydroxybenzoic acid (DHB) (Acros Organics, New Jersey, USA) at a concentration of 40 mg/mL in 70% methanol (*v*/*v*) under the following parameters: solvent sprayer was 50% methanol (*v*/*v*), dry gas temperature of 80 °C, 10 psi N_2_ gas pressure, flowrate of 0.1 mL/min, nozzle height at 65 mm with a speed of 1250 mm/min at 12 passes with 2 mm track spacing in crisscross (cc) pattern. DHB matrix was applied using an automatic sprayer with optimized parameters to ensure uniform coating and crystal formation across the entire tissue section, a strategy known to reduce spatial variability in ionization efficiency and mitigate ion suppression effects in plant tissues [51].

### 4.3. MALDI-TOF MSI and Ion Mobility Analysis

Spatial mapping of gingerols and shogaols was performed on a MALDI TOF SYNAPT XS High-Definition Mass Spectrometer (Waters Technologies Co., Ltd., Milford, MA, USA) equipped with an ion mobility spectrometer. The data were acquired in positive polarity at a mass range of 50–1200 Da with a spatial resolution of 100 µm using a laser energy of 300 arbitrary units (a.u.). For ion mobility spectrometry (IMS), the T-Wave parameters were set with a wave velocity of 650 m/s and a wave height of 40 V. Drift time acquisition was performed using a ramped wave velocity starting at 1385 m/s and decreasing to 440 m/s.

The acquisition pattern was defined using the HDImaging software (v1.5, Waters Technologies Co., Ltd., Milford, MA, USA) and was imported to MassLynx software (v4.2 Waters Technologies Co., Ltd., Milford, MA, USA) for data acquisition. External calibration was performed using red phosphorus (Sigma Aldrich, St. Louis, MO, USA) with a reference mass of *m*/*z* 464.6064. Raw data were then processed and analyzed using the HDImaging software. All MSI data were normalized to total ion count (TIC) to minimize variation in overall ionization efficiency across tissue regions [52] following the normalization strategy established in our previous MALDI MSI study [53]. Ion images were visualized using the ‘Weather1’ gradient, with no image smoothing applied and ‘Sqrt_Composition’ scale.

### 4.4. Toluidine Blue O Staining

Following the acquisition of tissue samples for MALDI MSI analysis, the succeeding ginger tissue samples were also prepared for histological staining. Cross-sections of the ginger rhizome were cryosectioned into 20 µm tissue sections and mounted on clean glass slides. The tissue sections were stained with 0.05% aqueous toluidine blue O (TBO) for 2 min. Excess stains were washed by flooding the sections with distilled water for 2 min until there was no excess stain around the sections. A drop of distilled water was added over the sections before mounting with a cover slip. The slides were examined using bright-field light microscopy. Three major regions of the rhizome were examined including the cortex, epidermis, and stele. Microscopic images were obtained for each region and documented.

### 4.5. UPLC-ESI-QTOF-MS and MS/MS Analysis

For comparative profiling, thicker sections (100 µm) were obtained from the embedded ginger rhizomes to increase material yield for extraction. Each sample was extracted with 5 mL LC-MS grade methanol, homogenized manually, sonicated for 5 min, and soaked for 4 h. After extraction, the samples were centrifuged to separate the supernatant.

Solid-phase extraction (SPE) was performed on the collected supernatant using Sep-Pak C18 cartridges (Waters Corp., Milford, MA, USA). The eluted extract was dried using a centrifugal evaporator (Genevac, SP Scientific, Ipswich, UK). The dried extract was then weighed and reconstituted to a concentration of 0.5 mg/mL in a 50% methanol (*v*/*v*) solution. Prior to LC-MS analysis, the solutions were filtered through 0.20 μm PTFE syringe filters (GE Healthcare Life Sciences, Chicago, IL, USA).

The metabolic profile of ginger methanolic extracts was analyzed using the Waters Acquity UPLC I-Class system (Waters Technologies Co., Ltd., Milford, MA, USA) coupled to a SYNAPT XS QTOF mass spectrometer, (Waters Technologies Co., Ltd., Milford, MA, USA) equipped with an ESI source. One microliter of the 0.5 mg/mL extract was injected into Acquity BEH C18 column (2.1 × 50 mm, 1.7 µm particle size) maintained at 40 °C.

The mobile phase system with a 0.2 mL/min flowrate was composed of water (A) and acetonitrile (B), both with 0.1% formic acid having a gradient profile ranging from 0 to 0.50 min, 20% B; 0.50 to 3.50 min, 20% to 40% B; 3.50 to 6.50 min, 40% to 70%B; 6.50 to 7.50 min, 70% to 100% B; 7.50 to 8.50 min, 100%B; 8.50 to 9.50 min, 100% to 20% B; 9.50 to 10.0 min, 20%B.

Mass spectra in the *m*/*z* range from 50 to 1200 were acquired at positive mode. The instrument was calibrated within the same mass range and polarity using an external calibrant, sodium formate (HCOONa) (Waters Corp., Milford, MA, USA). A lock-mass correction was performed using leucine-enkephalin (*m*/*z* 556.2771 [M+H]^+^) (Waters Corp., Milford, MA, USA). MS^E^ data (low energy, 0 eV; high energy, ramp 25 to 75 eV) were acquired in the continuum data format and a scan time set at 0.15 s.

Tandem mass spectrometry (MS/MS) analyses were performed using the same UPLC-ESI-QTOF-MS system and chromatographic conditions described above. Targeted MS/MS acquisition was carried out in positive ion mode for 6-, 8-, 10-gingerol, 6-, 8-, 10-shogaol, analyzed both as pure standards and in the sample extracts. The sodiated adduct ions [M+Na]^+^ were the predominant species observed and were therefore selected as precursor ions for fragmentation. Precursor ions were isolated with a mass window of ±0.5 Da and subjected to collision-induced dissociation using fixed collision energies optimized for each compound.

### 4.6. Statistical Analysis

#### 4.6.1. Data Processing and Principal Component Analysis

The UPLC-ESI-QTOF-MS data were processed using Progenesis QI software (Waters Corp., Milford, MA, USA). Spectral alignment, peak detection, deconvolution, and normalization were performed. Metabolic features were filtered using one-way analysis of variance (ANOVA) with a significance threshold of *p* ≤ 0.05, followed by fold change filtering (≥2). Features detected in blank samples were excluded, and only those with a minimum ion intensity of 10,000 were retained.

The resulting dataset was exported from Progenesis and imported into SIMCA (Sartorius Stedim Data Analytics AB, Umeå, Sweden). Unsupervised principal component analysis was carried out after Pareto scaling was applied.

#### 4.6.2. Hierarchical Clustering and Heatmap Visualization

A targeted dataset comprising signal intensities of 6-, 8-, and 10-gingerol and 6-, 8-, and 10-shogaol was extracted from the Progenesis output as [M+Na]^+^ adducts. These data were imported into MetaboAnalyst 6.0 for clustering and visualization.

Prior to analysis, the dataset was normalized by the sum of each sample’s intensities, followed by log10 transformation and Pareto scaling. Hierarchical clustering was performed using Euclidean distance and Ward’s linkage method. A heatmap was generated to visualize relative metabolite abundances and sample groupings across the accessions.

## 5. Conclusions

This study presents the first spatial metabolomics investigation of gingerols and shogaols in intact *Z. officinale* rhizome tissues using a multi-platform analytical approach. By integrating MALDI MSI with high-resolution histological imaging, ion mobility spectrometry, and UPLC-QTOF-MS and MS/MS, we developed a robust framework for mapping, identifying, and validating the tissue-specific distribution of specialized metabolites in ginger rhizome tissues.

Our results revealed that both gingerols and shogaols exhibit consistent accumulation within the cortex and stele regions of the rhizome, with minimal presence in the epidermis. These spatial patterns aligned with the distribution of oil cells and parenchyma observed via microscopy, supporting their role as key sites of metabolite storage. MALDI MSI enabled visualization of in situ chemical gradients, while ion mobility spectrometry successfully resolved isobaric overlaps between gingerol and shogaol ions, ensuring unambiguous compound identification directly within tissue sections. Complementary UPLC-QTOF-MS and MS/MS analysis confirmed the identities of six major gingerol and shogaol analogs and revealed significant inter-accession differences in their relative abundances. Untargeted PCA further highlighted metabolic distinctions among accessions, particularly for ginger H2 and H4, indicating broader biochemical divergence not captured through targeted analysis alone.

Collectively, the findings highlight the potential of combining spatial metabolomics with traditional analytical chemistry and histology to elucidate the intricate biochemical architecture of medicinal plants. The workflow established here provides a blueprint for metabolite localization studies that can be extended to other phytochemical classes and species. Beyond basic scientific insight, this work has practical implications for ginger breeding, phytochemical standardization, and targeted extraction, hence, enabling the selection of high-yielding chemotypes for functional foods, nutraceuticals, or pharmaceutical applications.

A limitation of this study is that MALDI MSI provides primarily qualitative spatial information and can be influenced by ion suppression and matrix effects, which may affect absolute quantitation across tissue regions. Moreover, while we focused on major gingerol and shogaol analogues, other bioactive metabolites, including minor phenolics and volatiles, were not annotated or analyzed downstream with MS/MS and dereplication.

Future work could extend on the comprehensive annotation of untargeted metabolites detected in this study, including minor phenolics and volatile constituents, to improve metabolome coverage and biological interpretation. Additionally, extending analyses to a broader range of accessions and developmental stages, including distinct rhizome maturity classes (e.g., immature, physiologically mature, and harvest-ready or marketable rhizomes), would enable stronger linkage of metabolite localization with genetic variation, agro-environmental conditions, and bioactivity.

Ultimately, this integrated approach opens new avenues for understanding plant biochemistry in a spatial context and accelerates the application of spatial metabolomics in plant-based health and industrial research.

## Data Availability

The raw data supporting the conclusion of this article will be made available by the authors, without undue reservation.

## Supplementary Materials

The following supporting information can be downloaded at https://www.mdpi.com/article/10.3390/molecules31040618/s1, Table S1. MALDI MS with ion mobility data of ginger rhizome cross-section from five ginger accessions (H1–H5). The table includes theoretical *m*/*z* and drift time values, along with observed *m*/*z* and drift times (dt) for each accession. Data are shown for each adduct observed for the six target compounds. Table S2. UPLC-ESI-QTOF-MS data of methanolic ginger extracts from five ginger accessions (H1–H5). The table includes theoretical *m*/*z* and retention time in minutes, along with observed *m*/*z* and retention time in minutes for each accession. Figure S1. MALDI ion images comparing five *Z. officinale* accessions (H1–H5). (A) Optical image of a tissue section from *Z. officinale* H1–H5. (B) Distribution of 8-gingerol [M+Na]^+^. (C) Distribution of 8-shogaol [M+Na]^+^. (D) Distribution of 10-gingerol [M+Na]^+^. (E) Distribution of 10-shogaol [M+Na]^+^. Ion images are normalized to total ion count, displayed in heatmap scale (red = 100%, violet = 0%), with tissue thickness of 20 µm, spatial resolution of 100 µm, and a scalebar of 12.94 mm. Figure S2. UPLC-ESI-QTOF-MS spectra of 6-, 8-, and 10-gingerols and 6-, 8-, and 10-shogaols acquired in ESI positive mode. Observed adducts for gingerols include [M−H_2_O+H]^+^, [M+Na]^+^, and [2M+Na]^+^, while shogaols were detected as [M+H]^+^ and [M+Na]^+^ ions. All observed *m*/*z* are within mass error of <5 ppm. Figure S3. UPLC-ESI-QTOF-MS/MS spectra using [M+Na]^+^ as the precursor ion. (A) 6-gingerol standard and 6-gingerol detected in the methanolic extract of ginger H1. (B) 6-shogaol standard and 6-shogaol detected in the methanolic extract of ginger H1. Figure S4. UPLC-ESI-QTOF-MS/MS spectra using [M+Na]^+^ as the precursor ion. (A) 8-gingerol standard and 8-gingerol detected in the methanolic extract of ginger H1. (B) 10-gingerol standard and 10-gingerol detected in the methanolic extract of ginger H1.
