# Supplementary material for: Spatial Profiling of Gingerol and Shogaol Analogues in Intact Zingiber officinale Rhizomes Using MALDI Mass Spectrometry Imaging
Source: Molecules. 2026 Feb 10;31(4):618. doi: 10.3390/molecules31040618 (PMC12943681; doi:10.3390/molecules31040618)
Supplement: Supplementary file 1 [file molecules-31-00618-s001.zip › molecules-3993181-supplementary.pdf]

Spatial Profiling of Gingerol and Shogaol Analogues in Intact *Zingiber officinale* Rhizomes Using MALDI Mass Spectrometry Imaging

Josie C. Torrecampo[1], Neaven Bon Joy M. Marcial[1], Chuckcris P. Tenebro[1], Janine J. Salcepuedes[1], Philip S. Cruz[2], Phil Aidan C. Cruz[2], Jonel P. Saludes[3,4], Doralyn S. Dalisay[1,5*]

1 Center for Chemical Biology and Biotechnology, University of San Agustin, Iloilo City 5000, Philippines; jtorrecampo@usa.edu.ph (J.C.T.); nbjmarcial@usa.edu.ph (N.B.J.M.M); ctenebro@usa.edu.ph (C.P.T.); jsalcepuedes@usa.edu.ph (J.J.S.)
2 Herbanext Laboratories, Inc., Bago City 6101, Philippines; philipcruz.herbanext@gmail.com (P.F.S.C.); aidancruz.herbanext@gmail.com (P.A.C.C.)
3 Center for Natural Drug Discovery and Development, University of San Agustin, Iloilo City 5000, Philippines; jsaludes@usa.edu.ph
4 Department of Chemistry, University of San Agustin, Iloilo City 5000, Philippines
5 Department of Biology, University of San Agustin, Iloilo City 5000, Philippines
* Correspondence: ddalisay@usa.edu.ph; Tel.: +63 33-501-0350

SUPPLEMENTARY INFORMATION

List of Supporting Tables

		Page
**Table S1**	MALDI MS with ion mobility data of ginger rhizome cross-section from five ginger accessions (H1–H5). The table includes theoretical *m/z* and drift time values, along with observed *m/z* and drift times (dt) for each accession. Data are shown for each adduct observed for the six target compounds.	**3**
**Table S2**	UPLC-ESI-QTOF-MS data of methanolic ginger extracts from five ginger accessions (H1–H5). The table includes theoretical *m/z* and retention time in minutes, along with observed *m/z* and retention time in minutes for each accession.	**4**

List of Supporting Figures

**Figure S1**	MALDI ion images comparing five *Z. officinale* accessions (H1–H5). (A) Optical image of a tissue section from *Z. officinale* H1–H5. (B) Distribution of 8-gingerol $[M+Na]^+$. (C) Distribution of 8-shogaol $[M+Na]^+$. (D) Distribution of 10-gingerol $[M+Na]^+$. (E) Distribution of 10-shogaol $[M+Na]^+$. Ion images are normalized to total ion count, displayed in heatmap scale (red = 100%, violet	**5**

= 0%), with tissue thickness of 20 μm, spatial resolution of 100 μm, and a scalebar of 12.94 mm.

Figure S2 UPLC-ESI-QTOF-MS spectra of 6-, 8-, and 10-gingerols and 6-, 8-, and 10-shogaols acquired in ESI positive mode. Observed adducts for gingerols include $(M-H_2O+H)^+$, $(M+Na)^+$, and $(2M+Na)^+$, while shogaols were detected as $(M+H)^+$ and $(M+Na)^+$ ions. All observed *m/z* are within mass error of < 5 ppm. **6**

Figure S3 UPLC-ESI-QTOF-MS/MS spectra using $[M+Na]^+$ as the precursor ion. (A) 6-gingerol standard and 6-gingerol detected in the methanolic extract of ginger H1. (B) 6-shogaol standard and 6-shogaol detected in the methanolic extract of ginger H1. **7**

Figure S4 UPLC-ESI-QTOF-MS/MS spectra using $[M+Na]^+$ as the precursor ion. (A) 8-gingerol standard and 8-gingerol detected in the methanolic extract of ginger H1. (B) 10-gingerol standard and 10-gingerol detected in the methanolic extract of ginger H1. **8**

Table S1. MALDI MS with ion mobility data of ginger rhizome cross-section from five ginger accessions (H1–H5). The table includes theoretical *m/z* and drift time values, along with observed *m/z* and drift times (dt) for each accession. Data are shown for each adduct observed for the six target compounds.

Compound	Theoretical (*m/z*, dt)	H1 (*m/z*, dt)	H2 (*m/z*, dt)	H3 (*m/z*, dt)	H4 (*m/z*, dt)	H5 (*m/z*, dt)
6-gingerol						
$[M]^+$	294.1831, 55.99	294.1833, 55.89	294.1838, 55.86	294.1832, 56.10	294.1833, 56.06	294.1830, 59.93
$[M+Na]^+$	317.1729, 58.46	317.1733, 58.19	317.1739, 58.18	317.1724, 58.48	317.1726, 58.40	317.1727, 58.23
$[M-H_2O]^+$	276.1725, 53.13	276.1739, 53.20	276.1732, 53.14	276.1723, 53.44	276.1722, 53.39	276.1725, 53.33
8-gingerol						
$[M]^+$	322.2144 ,63.45	322.2144, 63.59	322.2149, 63.56	322.2138, 63.85	322.2134, 63.82	322.2141, 63.67
$[M+Na]^+$	345.2042, 66.22	345.2049, 66.19	345.2051, 66.14	345.2049, 67.43	345.2040, 66.58	345.2036 ,66.42
$[M-H_2O]^+$	304.2038 ,59.95	304.2040, 59.75	304.2040, 59.93	304.2028, 60.05	304.2026, 59.93	304.2032, 59.82
10-gingerol						
$[M]^+$	350.2457, 70.73	350.2453, 70.79	350.2462, 70.78	350.2450, 70.96	350.2452, 71.01	350.2454, 70.87
$[M+Na]^+$	373.2355, 73.07	373.2353, 72.94	373.2366, 72.91	373.2356, 73.27	373.2354, 73.18	373.2356, 73.03
$[M-H_2O]^+$	332.2351, 66.46	332.2369, 66.15	332.2353, 66.65	332.2331, 66.81	332.2332, 66.80	332.2350, 66.33
6-shogaol						
$[M]^+$	276.1725 56.00	--	--	--	--	--
$[M+Na]^+$	299.1623, 60.11	299.1634, 59.86	299.1627, 59.86	299.1620, 60.01	299.1618, 60.03	299.1623, 59.93
8-shogaol						
$[M]^+$	304.2038, 63.87	--	--	--	--	--
$[M+Na]^+$	327.1936, 67.65	327.1936, 67.41	327.1938, 67.46	--	327.1931, 67.61	327.1948, 67.43
10-shogaol						
$[M]^+$	332.2351, 70.83	--	--	--	--	--
$[M+Na]^+$	355.2249, 72.23	355.2257, 72.13	355.2257, 72.12	--	355.2245, 72.15	355.2269, 71.22

Table S2. UPLC-ESI-QTOF-MS data of 6-, 8-, and 10-gingerols and 6-, 8-, and 10-shogaols acquired in ESI positive mode. Observed adducts for gingerols include $[M-H_2O+H]^+$, $[M+Na]^+$, and $[2M+Na]^+$, while shogaols were detected as $[M+H]^+$ and $[M+Na]^+$ ions. All observed *m/z* values are within mass error of < 5 ppm.

Compound	Theoretical (*m/z*, t_R)	H1 (*m/z*, t_R)	H2 (*m/z*, t_R)	H3 (*m/z*, t_R)	H4 (*m/z*, t_R)	H5 (*m/z*, t_R)
6-gingerol						
$[M-H_2O+H]^+$	277.1804, 5.37	277.1815, 5.38	277.1817, 5.39	277.1814, 5.39	277.1814, 5.39	277.1819, 5.39
$[M+Na]^+$	317.1729, 5.37	317.1739, 5.38	317.1740, 5.39	317.1736, 5.39	317.1741, 5.39	317.1737, 5.39
$[2M+Na]^+$	611.3560, 5.37	611.3586, 5.38	611.3590, 5.38	611.3585, 5.39	611.3580, 5.39	611.3586, 5.39
8-gingerol						
$[M-H_2O+H]^+$	305.2117, 6.59	305.2121, 6.61	305.2123, 6.61	305.2122, 6.61	305.2120, 6.61	305.2117, 6.61
$[M+Na]^+$	345.2042, 6.59	345.2040, 6.61	345.2044, 6.61	345.2048, 6.61	345.2047, 6.61	345.2044, 6.61
$[2M+Na]^+$	667.4186, 6.59	667.4196, 6.61	667.4202, 6.61	667.4198, 6.61	667.4194, 6.61	667.4178, 6.61
10-gingerol						
$[M-H_2O+H]^+$	333.2430, 7.65	333.2433, 7.66	333.2438, 7.67	333.2435, 7.67	333.2435, 7.66	333.2436, 7.66
$[M+Na]^+$	373.2355, 7.65	373.2364, 7.66	373.2363, 7.67	373.2361, 7.67	373.2359, 7.66	373.2360, 7.66
$[2M+Na]^+$	723.4812, 7.65	723.4837, 7.66	723.4839, 7.67	723.4833, 7.67	723.4838, 7.66	723.4833, 7.66
6-shogaol						
$[M+Na]^+$	299.1623, 6.82	299.1628, 6.8	299.1626, 6.83	299.1631, 6.83	299.1617, 6.83	299.1616, 6.84
8-shogaol						
$[M+Na]^+$	327.1936, 7.84	327.1926, 7.86	327.1927, 7.86	327.1929, 7.86	327.1931, 7.87	327.1918, 7.86
10-shogaol						
$[M+Na]^+$	355.2249, 8.44	355.2265, 8.45	355.2265, 8.45	355.2261, 8.45	355.2263, 8.46	355.2269, 8.45

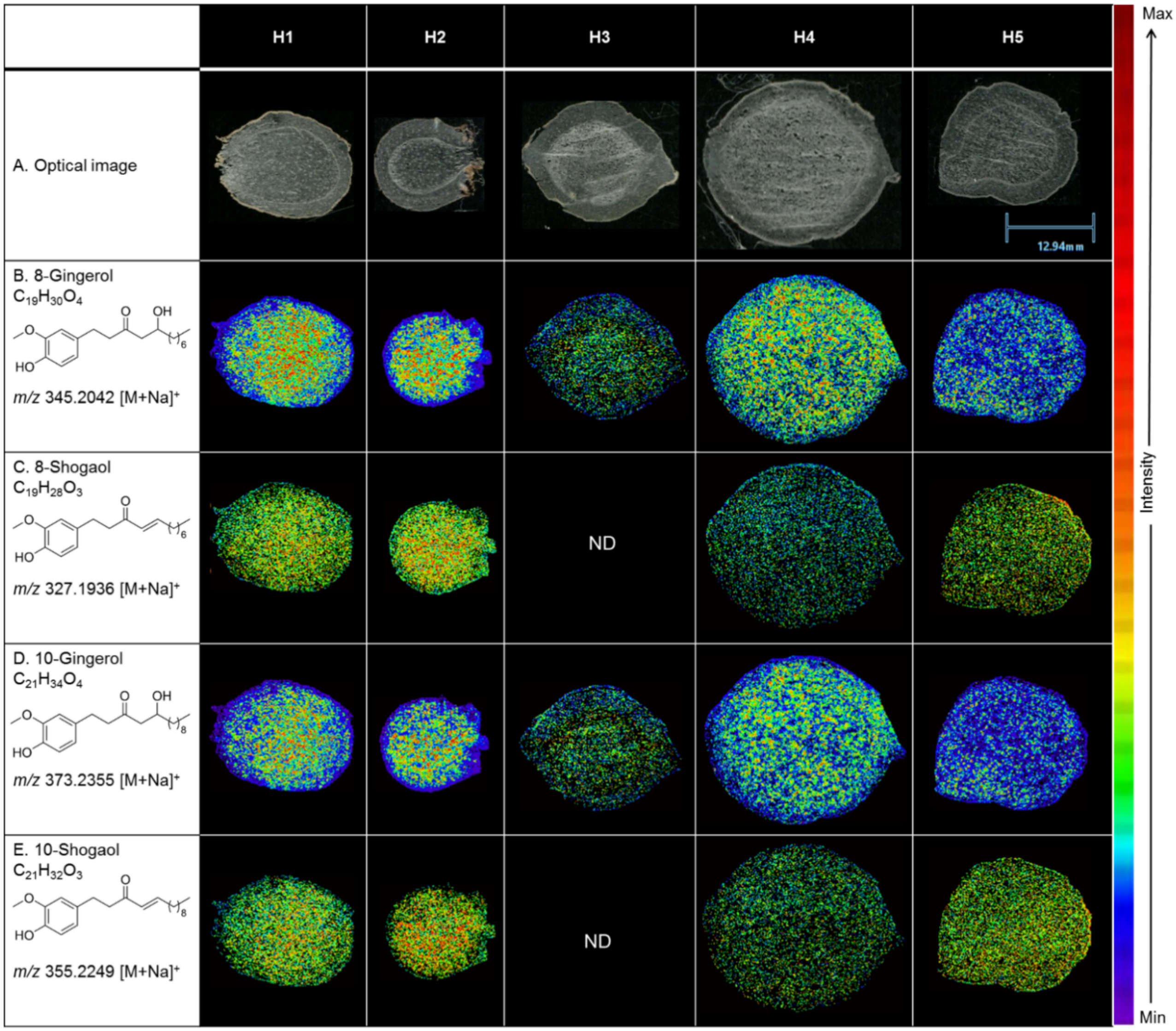

Figure S1. MALDI ion images comparing five *Z. officinale* accessions (H1–H5). (A) Optical image of a tissue section from *Z. officinale* H1–H5. (B) Distribution of 8-gingerol $[M+Na]^+$. (C) Distribution of 8-shogaol $[M+Na]^+$. (D) Distribution of 10-gingerol $[M+Na]^+$. (E) Distribution of 10-shogaol $[M+Na]^+$. Ion images are normalized to total ion count, displayed in heatmap scale (red = 100%, violet = 0%), with tissue thickness of 20 μm, spatial resolution of 100 μm, and a scalebar of 12.94 mm.

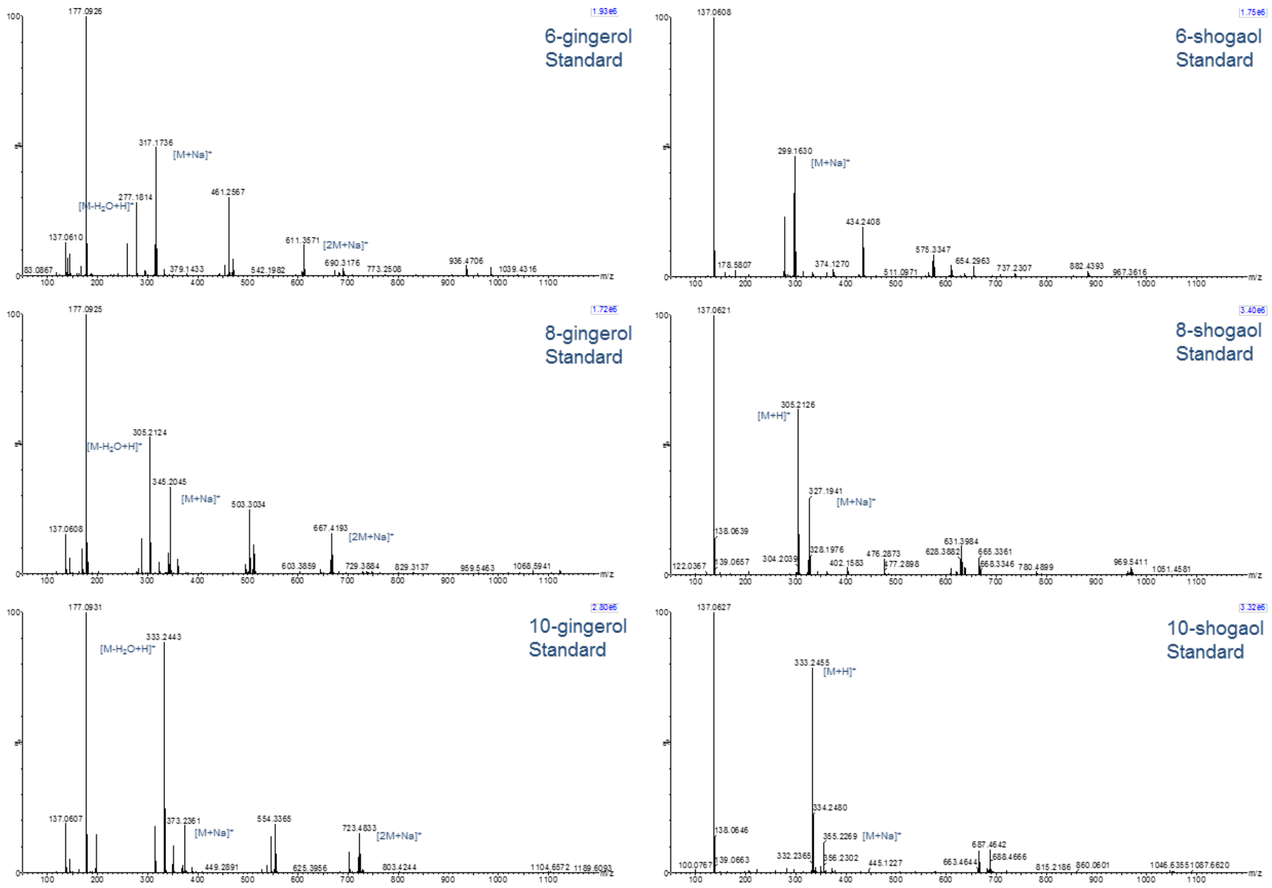

Figure S2. UPLC-ESI-QTOF-MS spectra of 6-, 8-, and 10-gingerols and 6-, 8-, and 10-shogaols acquired in ESI positive mode. Observed adducts for gingerols include $[M-H_2O+H]^+$, $[M+Na]^+$, and $[2M+Na]^+$, while shogaols were detected as $[M+H]^+$ and $[M+Na]^+$ ions. All observed *m/z* values are within mass error of < 5 ppm.

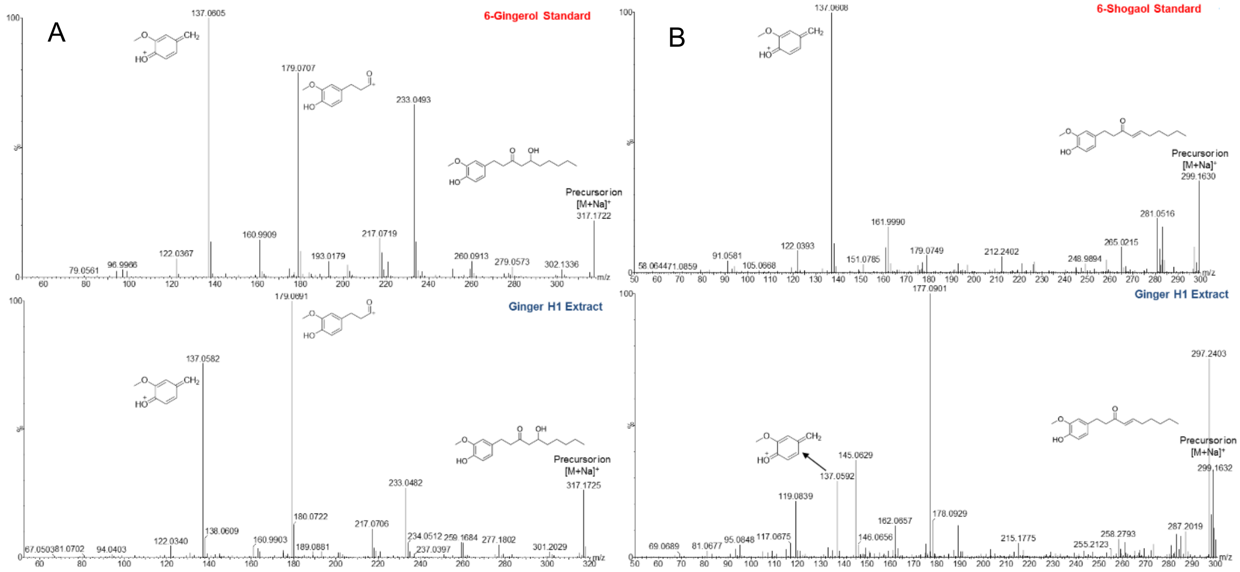

Figure S3. UPLC-ESI-QTOF-MS/MS spectra using $[M+Na]^+$ as the precursor ion. (A) 6-gingerol standard and 6-gingerol detected in the methanolic extract of ginger H1. (B) 6-shogaol standard and 6-shogaol detected in the methanolic extract of ginger H1.

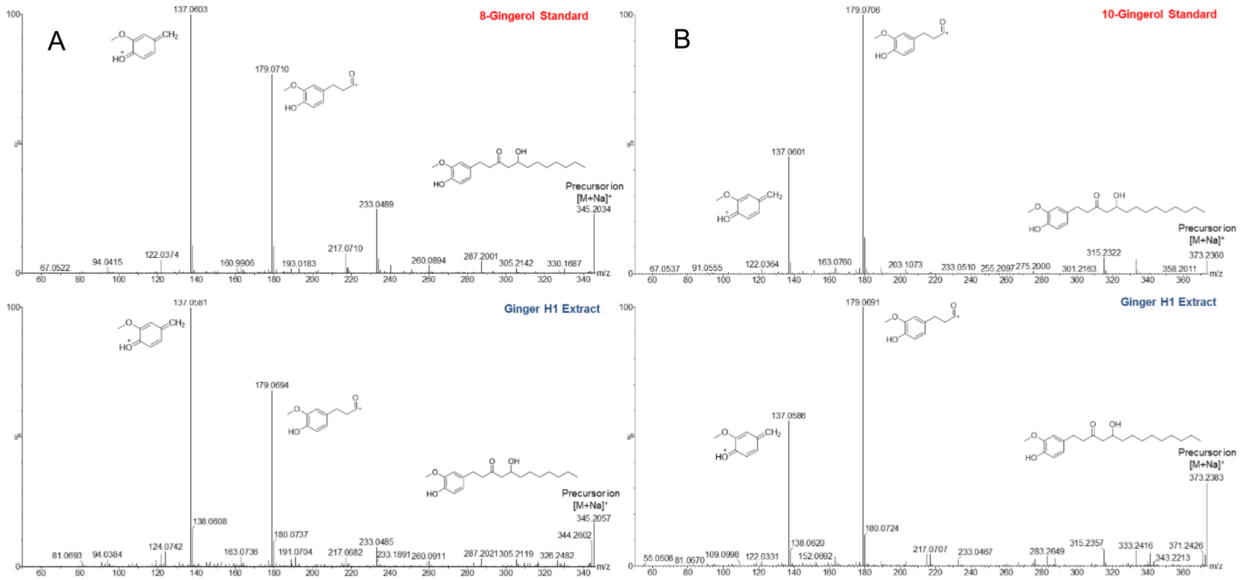

Figure S4. UPLC-ESI-QTOF-MS/MS spectra using $[M+Na]^+$ as the precursor ion. (A) 8-gingerol standard and 8-gingerol detected in the methanolic extract of ginger H1. (B) 10-gingerol standard and 10-gingerol detected in the methanolic extract of ginger H1.
